# Draft Genome Sequence of Staphylococcus chromogenes ATCC 43764, a Coagulase-Negative Staphylococcus Strain with Antibacterial Potential

**DOI:** 10.1128/MRA.00492-21

**Published:** 2021-06-03

**Authors:** Denny Chin, Shayna R. Deecker, Alexander W. Ensminger, David E. Heinrichs

**Affiliations:** a Department of Microbiology and Immunology, University of Western Ontario, London, Ontario, Canada; b Department of Biochemistry, University of Toronto, Toronto, Ontario, Canada; c Department of Molecular Genetics, University of Toronto, Toronto, Ontario, Canada; University of Rochester School of Medicine and Dentistry

## Abstract

Staphylococcus chromogenes can cause subclinical mastitis in cows, and some strains have also demonstrated antibacterial activity against pathogens such as methicillin-resistant Staphylococcus aureus (MRSA). Here, we report the draft genome sequence of the *S. chromogenes* type strain ATCC 43764, which secretes the prodrug 6-thioguanine (6-TG), which antagonizes MRSA virulence.

## ANNOUNCEMENT

Staphylococcus chromogenes is a bovine pathogen that causes subclinical mastitis (1). Interestingly, *S. chromogenes* is known to colonize the same niches in animals as the human pathogen Staphylococcus aureus, and several *S. chromogenes* strains have been noted to have antibacterial activity against S. aureus (2, 3). Indeed, we recently showed that *S. chromogenes* strain ATCC 43764, which was isolated from pig skin, antagonizes S. aureus virulence through the production of the prodrug 6-thioguanine (6-TG). Moreover, we identified the genetic basis for 6-TG biosynthesis in this strain (4). Here, we report the genome sequence of *S. chromogenes* ATCC 43764 to provide insight into *S. chromogenes* genetics and to provide information on the 6-TG biosynthetic cluster in this strain.

We obtained *S. chromogenes* ATCC 43764 from the American Type Culture Collection (ATCC) and grew this strain on tryptic soy agar (TSA) at 37°C overnight. Single colonies were inoculated into tryptic soy broth (TSB) and grown at 37°C overnight with shaking at 200 rpm. Genomic DNA was isolated by phenol-chloroform extraction (5). The extracted genomic DNA was tagmented using the Nextera tagmentation kit (Illumina) with a modified protocol (6). The tagmented products were sequenced on an Illumina NextSeq 550 instrument with paired-end sequencing (2 × 150 bp) at the Microbial Genome Sequencing Center (MiGS; https://www.migscenter.com/) in Pittsburgh, PA. The DNA sequence reads were trimmed using Trimmomatic version 0.36 (7) with the following parameters: ILLUMINACLIP, NexteraPE:2:30:10:8:true; LEADING, 20; TRAILING, 20; SLIDINGWINDOW, 4:20; MINLEN, 36. The reads were assembled using the SPAdes version 3.13 assembler (8) in careful mode, as a plugin in Geneious Prime. The contigs were initially annotated using Prokka version 1.12 (9), and the final draft genome sequence was autoannotated at NCBI using the Prokaryotic Genome Annotation Pipeline (PGAP). The whole-genome average nucleotide identity (ANI) of the *S. chromogenes* ATCC 43764 draft genome sequence was calculated using FastANI version 1.32 (10) with default settings. The draft genome sequence was compared to four completed *S. chromogenes* genome sequences from NCBI, and the ANI estimate was ∼98% for each pairwise comparison (Table 1).

**TABLE 1:** Average nucleotide identity estimate for *S. chromogenes* ATCC 43764 compared to completed *S. chromogenes* genomes from NCBI

Query isolate	Reference isolate	Reference isolate GenBank accession no.	ANI estimate (%)
*S. chromogenes* ATCC 43764	*S. chromogenes* 17A	CP031274.1	98.3011
*S. chromogenes* ATCC 43764	*S. chromogenes* 20B	CP031471.1	99.2226
*S. chromogenes* ATCC 43764	*S. chromogenes* 34B	CP031470.1	98.0153
*S. chromogenes* ATCC 43764	*S. chromogenes* 1401	CP046028.1	98.2645

In total, 4,113,428 Illumina reads (BioSample accession number SAMN14848453) were assembled into 77 contigs. After size filtering (excluding contigs less than 200 bp), the final draft genome consisted of 34 contigs. The *N*_50_ value of this assembly is 250,031 bp, the genome coverage is ∼237×, and the GC content of the genome is 36.6%. The chromosome of *S. chromogenes* ATCC 43764 is 2,274,307 bp. Based on PGAP, the numbers of predicted coding sequences and tRNA operons in the genome were 2,154 and 55, respectively. The searchable protein IDs assigned to the 6-TG synthesis operon are MBP0047068.1, MBP0047069.1, MBP0047070.1, MBP0047071.1, MBP0047072.1, and MBP0047073.1.

In conclusion, a 6-TG-producing strain of Staphylococcus chromogenes was characterized. This is evidence that genetic information encoding novel small molecules with antimicrobial properties continues to be identified by sequencing additional bacterial genomes from the rich reservoir of microbes that inhabit humans and other mammals. The draft genome sequence of *S. chromogenes* ATCC 43764 will be useful for identifying the 6-TG biosynthetic operon in other strains and species and for future taxonomic studies for comparative genomics.

### Data availability.

The raw reads have been submitted to the NCBI Sequence Read Archive (SRA) database under the BioProject accession number PRJNA630769 and specifically the BioSample accession number SAMN14848453. This whole-genome shotgun project has been deposited at DDBJ/ENA/GenBank under the accession number JAGIPW000000000. The version described in this paper is the first version, JAGIPW010000000. The publicly available genome data records for the reference genomes referred to in Table 1 are accessible under the following accession numbers: CP031274.1, CP031471.1, CP031470.1, and CP046028.1. 
